# Welcome to *FASEB BioAdvances*, the new open access journal by the Federation of American Societies for Experimental Biology (FASEB)

**DOI:** 10.1096/fba.2018-00077

**Published:** 2019-01-03

**Authors:** Jasna Markovac

**Affiliations:** ^1^ CalTech Pasadena California

Although the idea of starting an open access journal had been discussed for years, the need for it became clear at FASEB's Publications and Communications Committee meeting in March 2016. The increased uptake of open access publishing in *The FASEB* *Journal *made it evident that there was strong and growing demand for this type of publishing among authors and their funders, and that this demand was likely not being met fully by *The FASEB Journal's *hybrid open access approach. Furthermore, given *The FASEB Journal's *selectivity based on publishing priority, not all studies will be published, regardless of how well the experiments are designed and conducted. This leaves a number of authors and potential authors seeking to publish multi/transdisciplinary work with FASEB for whom *The FASEB* *Journal *may not be the best fit. Finally, as the focus toward reproducibility intensifies, FASEB wanted to provide a venue where scientists can publish reports covering their ability (or inability) to replicate published experiments. Because these reports lack the novelty of new experimentation, they would likely never be published in *The FASEB* *Journal*, despite their critical importance to working scientists.

Building on the invaluable experience of FASEB*'*s constituent societies in starting their own open access publications, we planned a new journal under the FASEB brand, with the primary goal of publishing well‐designed, sound science and reproducibility reports that are multi/transdisciplinary, without regard for any perceived citation impact. Doing this is critical to FASEB*'*s mission of supporting the advancement of the biological sciences both in the United States and around the globe. “Publish or perish” is as true today as it was many decades ago, but also has the added pressures of securing diminishing sources for research funding and navigating the ever‐increasing modes of publishing made possible through technology. As someone who started in academia, then moved to commercial publishing, and is now back in academia, I believe that academic, and especially society, publishers have a responsibility to the communities they serve to make the process of publishing research easier, more straightforward, and ultimately more effective. My hope is that in time, *FASEB BioAdvances *will achieve these lofty goals.

On a personal note, I would like to say that seeing *FASEB BioAdvances *come to fruition from an editor*'*s perspective has been eye‐opening, fulfilling, and a lot of fun. During my career as a researcher, I published papers in scientific journals. When I worked for publishing companies, I was responsible for individual journals, as well as for entire journal programs. I knew how to run journals, keep them on schedule, and manage the “back end” functions. Serving as an editor has been an entirely different experience. From the initial discussions about the possibility of launching an OA journal, to this past June when we first began taking submissions, and now, to the publication of the first issue, I see how launching a publication like *FASEB BioAdvances *is truly a team effort. I think this first issue is a good example of how FASEB can help the research community communicate its work. I hope you enjoy reading the journal and would encourage you to submit your work to us.

